# Radiation Exposure Perturbs IL-17RA-Mediated Immunity Leading to Changes in Neutrophil Responses That Increase Susceptibility to Oropharyngeal Candidiasis

**DOI:** 10.3390/jof8050495

**Published:** 2022-05-10

**Authors:** Jessica Saul-McBeth, John Dillon, Dylan Launder, Maura Hickey, Elise Mein-Chiain Yi, Yusuf Daboul, Priosmita Biswas, Elahheh Salari, E. Ishmael Parsai, Heather R. Conti

**Affiliations:** 1 Department of Biological Sciences, University of Toledo, Toledo, OH 43606, USA; jessica.saul@utoledo.edu (J.S.-M.); john.dillon@rockets.utoledo.edu (J.D.); dylan.launder@rockets.utoledo.edu (D.L.); maura.hickey@rockets.utoledo.edu (M.H.); elise.yi@rockets.utoledo.edu (E.M.-C.Y.); yusuf.daboul@rockets.utoledo.edu (Y.D.); priosmita.biswas@rockets.utoledo.edu (P.B.); 2 Department of Radiation Oncology, Division of Medical Physics, The University of Toledo, Toledo, OH 43606, USA; elahheh.salari@rockets.utoledo.edu (E.S.); e.parsai@utoledo.edu (E.I.P.)

**Keywords:** oral immunity, interleukin-17, candidiasis, neutrophil, antifungal

## Abstract

Fungal infections caused by *Candida albicans* are a serious problem for immunocompromised individuals, including those undergoing radiotherapy for head and neck cancers. Targeted irradiation causes inflammatory dysregulation and damage to the oral mucosa that can be exacerbated by candidiasis. Post-irradiation the cytokine interleukin-17 (IL-17) protects the oral mucosae by promoting oral epithelial regeneration and balancing the oral immune cell populations, which leads to the eventual healing of the tissue. IL-17 signaling is also critical for the antifungal response during oropharyngeal candidiasis (OPC). Yet, the benefit of IL-17 during other forms of candidiasis, such as vulvovaginal candidiasis, is not straightforward. Therefore, it was important to determine the role of IL-17 during OPC associated with radiation-induced inflammatory damage. To answer this question, we exposed *Il17ra*^−/−^ and wild-type mice to head-neck irradiation (HNI) and OPC to determine if the IL-17 signaling pathway was still protective against *C. albicans*. HNI increased susceptibility to OPC, and in *Il17ra^−/−^* mice, the mucosal damage and fungal burden were elevated compared to control mice. Intriguingly, neutrophil influx was increased in *Il17ra^−/−^* mice, yet these cells had reduced capacity to phagocytose *C. albicans* and failed to clear OPC compared to immunocompetent mice. These findings suggest that radiotherapy not only causes physical damage to the oral cavity but also skews immune mediators, leading to increased susceptibility to oropharyngeal candidiasis.

## 1. Introduction

*Candida albicans (C.albicans)* is a common fungal commensal of the human microbiota that colonizes the orogastrointestinal and reproductive mucosa of 50–80% of healthy individuals [1]. *C. albicans* is the main cause of oropharyngeal candidiasis (OPC), which is common in infants, the elderly, denture wearers, and patients on antibiotics or corticosteroids. OPC is a complication for many immunocompromised populations, including individuals with HIV/AIDS [2]. Patients receiving head and neck irradiation (HNI), with or without combination chemotherapy, are also prone to OPC [3,4,5]. Additionally, the loss of mucosal barrier integrity post-irradiation can allow for infections that breach the mucosae and cause disseminated infections [6,7] Systemic, blood-borne nosocomial infections attributed to *C. albicans* are associated with mortality rates as high as 40% in some patient groups [8].

It is well-established that the proinflammatory cytokine interleukin-17 (IL-17) is a central mediator in oral immune responses to *C. albicans* in both humans and mice [9,10,11,12]. During OPC activated innate TCRβ+ ‘natural’ T-helper 17 (Th17) cells and γδ-T cells, along with adaptive Th17 cells secrete IL-17A (IL-17), IL-17F, and IL-22 [11,13]. These cytokines cooperatively upregulate neutrophil chemokines, antimicrobial proteins (AMPs), and pro-inflammatory cytokines, which together protect against candidiasis [11,14]. IL-17 and IL-17F signal through the IL-17 receptor (IL-17R), composed of the IL-17RA and IL-17RC subunits that are highly expressed on epithelial and stromal cells [9]. IL-17R is essential for immunity to OPC, whereas *Il17ra*^−/−^ and *Il17rc*^−/−^ mice are highly susceptible to oral mucosal *Candida* infection [13,14,15].

While IL-17 is considered protective against OPC, the cytokine plays diverse roles in immunity and inflammation [16]. The Th17/IL-17 axis is considered pathogenic in many autoimmune conditions, including rheumatoid arthritis and psoriasis [17,18,19]. Yet, in other inflammatory conditions, particularly in the gut, IL-17 is beneficial to barrier maintenance via the regulation of tight-junction proteins and by promoting epithelial proliferation [20,21]. Even in different forms of candidiasis the role of IL-17 is not as straightforward as it is in OPC [22]. In one example of experimental vulvovaginal candidiasis (VVC), IL-17 was dispensable for protection against infection [23]. This aligns with data from humans where IL-17 deficiency does not lead to increased susceptibility to VVC [24]. Yet, others have shown that IL-17 is beneficial against *Candida* in the vaginal tract [25]. It is generally thought that a strong neutrophil response is pathogenic in VVC, regardless of whether recruitment is IL-17-driven or not [26,27]. Since radiation exposure leads to a large number of neutrophils in the oral tissue during IL-17RA deficiency, it was important to determine the phenotype of these neutrophils during OPC associated with radiation damage [28].

The damage radio- and chemotherapy cause to the mucosal layer of the orogastrointestinal tract is called mucositis [29,30]. Oral mucositis (OM) can be a severe complication of HNI, associated with unmanageable pain and a high financial burden that oftentimes can lead to the patient discontinuing treatment for the malignancy [3,31]. Radiation exposure to the head and neck regions of mice induces IL-17 expression in the oral mucosa, with the highest levels of the cytokine detected during peak OM damage [28]. *Il17ra*^−/−^ mice exposed to HNI suffer excessive OM damage because IL-17 supports oral epithelial regeneration and regulates the neutrophil response, whereas, in the absence of IL-17RA, other cytokines including IL-1 are dysregulated, leading to increased neutrophilic infiltration and inflammatory damage [28]. As noted above, various patient populations are susceptible to oral *Candida* infections, yet it is not fully understood how different forms of immunosuppression affect Th17-related immunity directly [10]. Therefore, it was critical to experimentally address the question of how irradiation-induced damage to the oral mucosa leads to OPC susceptibility in the context of IL-17RA deficiency. To this end, we exposed *Il17ra*^−/−^ and wild-type (WT) mice to HNI and OPC in order to determine if the IL-17 signaling pathway was still protective against *Candida*, even when oral mucosal damage is exacerbated by radiation. We found that the presence of *C. albicans* after HNI led to elevated levels of *Il17a* transcript in tongue tissue compared to mice exposed only to the fungus. Then, in the absence of IL-17RA, damage and fungal burden were elevated after irradiation and infection compared to control mice. Neutrophil levels were higher in *Il17ra*^−/−^ mice after HNI and OPC, yet these neutrophils were not protective and failed to clear *Candida* as the cells normally would during OPC in an immunocompetent mouse. The neutrophils from *Il17ra*^−/−^ mice exhibited aberrant activation properties and reduced capacity to phagocytose *C. albicans* compared to WT mice. Altogether, IL-17 signaling is required to avoid neutrophil-driven immunopathology in the setting of radiation-induced OPC.

## 2. Materials and Methods

### 2.1. Mice

Mice were acquired by materials transfer agreement (MTA) with Amgen (*Il17ra*^−/−^) [32]. In all experiments, age- and gender-matched littermate controls or C57BL/6 WT controls (The Jackson Laboratory, Inc., Bar Harbor, ME, USA) were used. All mice were housed with food and water ad libitum under a 12 h dark/light cycle in a specific pathogen-free facility at the University of Toledo.

### 2.2. Radiation Induced OM

Mice were exposed to HNI as previously described in [28]. Briefly, mice were immobilized using an anesthesia protocol approved by the Department of Laboratory Animal Research at the University of Toledo. Mice were aligned in the radiation field under a linear accelerator to deliver 22.5 Gy using a 6 MeV electron beam at the rate of 1000 cGy/min in a single fraction directly to the head and neck region of the mice. Following irradiation, animals were removed and housed in a climate and light/dark controlled environment and allowed free access to food and water. Animals were monitored daily for changes in weight and activity.

### 2.3. Candida albicans Culturing and Handling

*Candida albicans* SC5314 was cultured in YPD by standard methods [33]. Colonies were grown overnight in YPD broth, and the concentration of *Candida* yeast cells were adjusted the following morning for infection of mice.

### 2.4. Murine Model of Oropharyngeal Candidiasis

OPC was induced 12–15 h after HNI by sublingual inoculation with a preweighed cotton ball saturated in *C. albicans* (SC5314) for 75 min under anesthesia as previously described in [34]. On Day 2 or 4 following infection, mice were euthanized, and tongues, kidney, stomachs, and intestines were extracted to determine tissue fungal burden.

### 2.5. Macroscopic and Histopathologic Examination

Tongues were rinsed with PBS and stained with 1% toluidine blue for 2 min, followed by washing with acetic acid for 30 s to reveal ulcerative lesions as previously described [28]. The percentage of toluidine blue-positive areas were calculated using ImageJ software and % damage quantified by the area of toluidine blue positive area/surface area of whole tongue *100. Tissues were formalin-fixed, paraffin-embedded, and sectioned at a thickness of 5 µm. Ulcer size, mucosal thickness, and cellular infiltrate were measured in H&E-stained tissue using an EVOS FLc microscope (Thermo Fisher Scientific, Inc., Waltham, MA, USA). Evaluators blinded to the treatment group and mouse cohort analyzed the tissue sections.

### 2.6. Immunohistochemistry

Tissues sections were dehydrated with xylene and ethanol gradient, and antigen retrieval and blocking performed. Sections were further labeled with MPO (R&D Systems, Minneapolis, MN, USA). Secondary biotinylated antibody was applied, and slides were incubated at room temperature for 1 h. Signals were detected using Sigma Fast tablets to make the DAB solution (Sigma Aldrich, St. Louis, MO, USA), and the reaction was stopped by placing slides in TBS. For occludin (Santa Cruz Biotechnology, Santa Cruz, CA, USA), and CD63 (R&D Systems, Minneapolis, MN, USA) IHC was performed on paraffin sections using avidin-biotin-peroxidase complex (streptavidin–biotin labeled method) with the Cell and Tissue staining kit (R&D Systems, Minneapolis, MN, USA). The manufacturer’s protocol was followed. For periodic acid-Schiff staining (PAS) of *Candida albicans*, slides were dehydrated and stained according to manufacturer’s protocol (Sigma-Aldrich, Inc., Burlington, MA, USA).

### 2.7. Complete Blood Count

EDTA anti-coagulated blood samples from cardiac puncture were used to obtain a complete blood count with an Insight V5 Hematology Analyser (Woodley Equipment, Bolton, Lancashire, UK).

### 2.8. Realtime PCR

Total RNA was extracted using TRI reagent (Sigma-Aldrich, St. Louis, MO, USA) and RNA (1 µg) reverse-transcribed by High-Capacity cDNA RT kit (Thermo Fisher Scientific, Waltham, MA, USA) at 25 °C for 10 min, 37 °C for 120 min, followed by 85 °C for 5 min. Quantitative PCR was performed using PowerUp SYBR green Master Mix and a Quant Studio 3 detection system (Applied Biosystems, Waltham, MA, USA), as specified by the manufacturer. The crossing point was defined as the maximum of the second derivative from the fluorescence curve. For quantification, relative mRNA expression of specific genes using the 2^−ΔCT^ method and GAPDH housekeeping gene for normalization was used. Primers for *Defb3* and *Mmp9* were QuantiTect Primer assays from Qiagen’s pre-made primer library (QIAGEN, Germantown, MD, USA). The remaining primers were PrimeTime qPCR Primer assays (IDT Integrated DNA Technologies, Coralville, IA, USA). Assays were performed in biological triplicate in technical triplicate.

### 2.9. Flow Cytometry

Tongue tissue was mechanically homogenized in RPMI 1640 media then incubated at 37 °C for 42 min on a GentleMACS Dissociator (Miltenyi Biotec, LLC, Auburn, CA, USA) using Tissue Dissociation Kits (Miltentyi Biotec, LLC, Auburn, CA, USA) then passed through a 40 μm cell strainer to form single-cell suspensions. After a brief centrifugation, cells were reconstituted with PBS supplemented with 2% FBS and 2 mM EDTA. The 1 × 10^6^/mL viable cells were obtained by staining with trypan blue and counting on a hemocytometer. For analysis of neutrophils, an initial incubation of CD16/CD32 Fc Block (BD Biosciences, Franklin Lakes, NJ, USA) was followed by staining with the following antibodies, all from BioLegend, Inc. (San Diego, CA, USA): CD63-PE (NVG-2), CD11b-PerCP/Cyanine5.5 (M1/70), LFA-1-PE/Cyanine7 (H155-78), and GR-1-APC (RB6-8C5), along with Sytox Blue for cell viability. Analysis of *Candida* phagocytosis was performed using a GFP-expressing *C. albicans* strain (SGH275, generously provided by Aaron Mitchell, University of Georgia). Flow cytometry was performed on an LSRFortessa (BD Biosciences, Franklin Lakes, NJ, USA).

Analyses of flow cytometry results were performed using FlowJo (BD Biosciences). Gating strategy can be found in Figure S1. Initially, doublets were removed by gating on FSC-A versus (vs.) FSC-H. Viable cells were then obtained using a negative Sytox Blue signal, followed by a general leukocyte gate using FSC-A vs. SSC-A. Neutrophils were acquired by gating on either SSC-A vs. GR-1 positive signal or CD11b vs. GR-1 double positive signal, followed by individual histogram analysis of CD11b-positive cells and CD63-positive cells. Finally, the histogram analysis of GFP-positive cells is used to determine the number of activated neutrophils positive for *C. albicans* phagocytosis. Unstained tongue cells and cells from sham mice were used for determining relative positive fluorescent signals.

### 2.10. Statistics

At least three biological replicates were performed for all experiments and experiments were repeated at least two times, as specified in the figure legends. Normally distributed data were analyzed via ANOVA with Tukey’s post hoc analysis or Student’s *t* test. Nonparametric data were analyzed by Kruskal–Wallis or Mann–Whitney using GraphPad Prism (V8.4.3) as indicated in the Figure legends. *p* values < 0.05 were considered significant.

## 3. Results

### 3.1. Exposure to Candida albicans after HNI Leads to Development of Severe OPC

**Figure 1:**
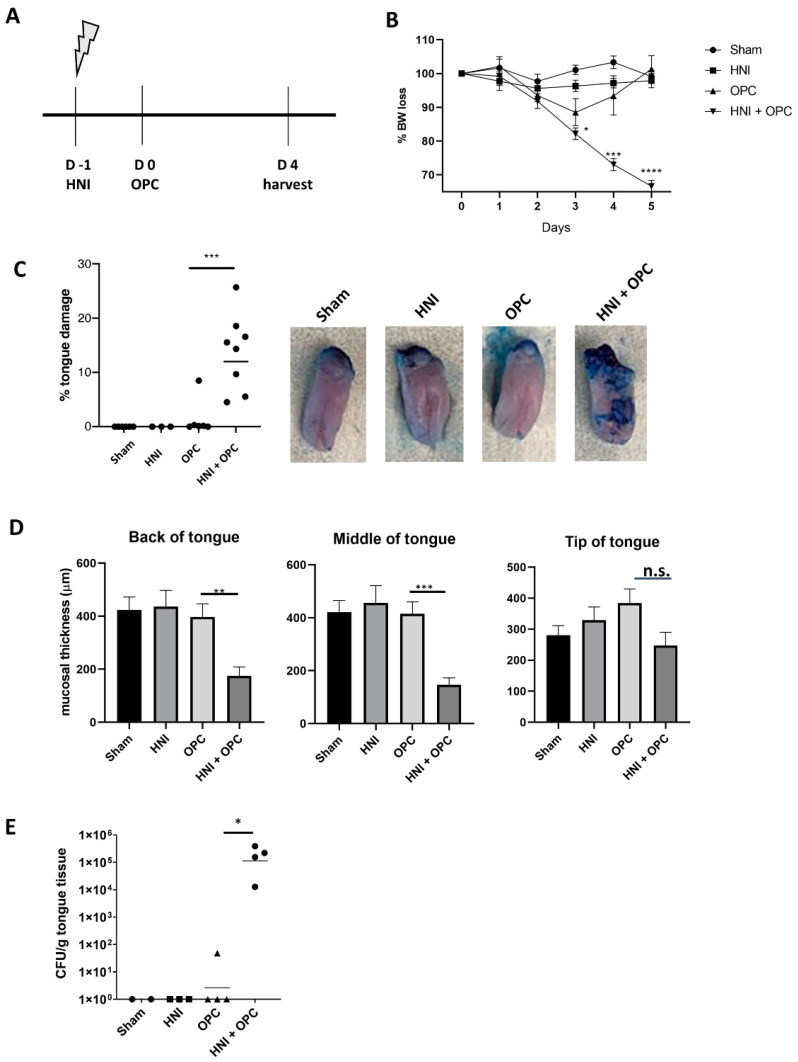
HNI increases susceptibility to OPC. (**A**) Timeline of HNI and OPC exposure. Mice were exposed to HNI on day -1 and OPC on day 0, 12–15 h after HNI exposure. WT mice received no radiation and no OPC (sham), HNI alone (HNI), OPC alone (OPC), or both (HNI + OPC). Tongues were harvested on Day 4. (**B**) HNI + OPC mice (n = 9) lost significantly more body weight (BW) compared to Sham (n = 4), HNI-only (n = 7), OPC-only (n = 7) groups. All mice were weighed daily, and % body weight loss (grams) on each day of infection determined. Analyzed by one-way ANOVA with Tukey’s post hoc. (**C**) The portions of the tongue with visual lesions were quantified by determining the surface area of the tongue positive for staining with toluidine blue compared to the total surface area of tongue on each mouse on Day 4 post infection using ImageJ software. (**D**) Quantification of mucosal thickness on the tip, middle, and back of tongue. Measured from basal stem cell layer up to papillae using ImageJ software. Three mice were analyzed from each group, with three sections per mouse. Investigators analyzing toluidine blue staining and measurement of mucosal thickness were blinded to treatment and mouse cohort. Analyzed by one-way ANOVA with Tukey’s post hoc. (**E**) WT mice were subjected to HNI + OPC and on Day 4 tongue tissue was harvested, tissue homogenized and plated then CFU/g of tongue tissue was determined in triplicate. Analyzed by Mann-Whitney U test. Data shown as geometric mean. (* *p* < 0.05, ** *p* < 0.01, *** *p* < 0.001, **** *p* < 0.0001). n.s. is not significant. Data represent 3 experimental repeats.

**Figure 2:**
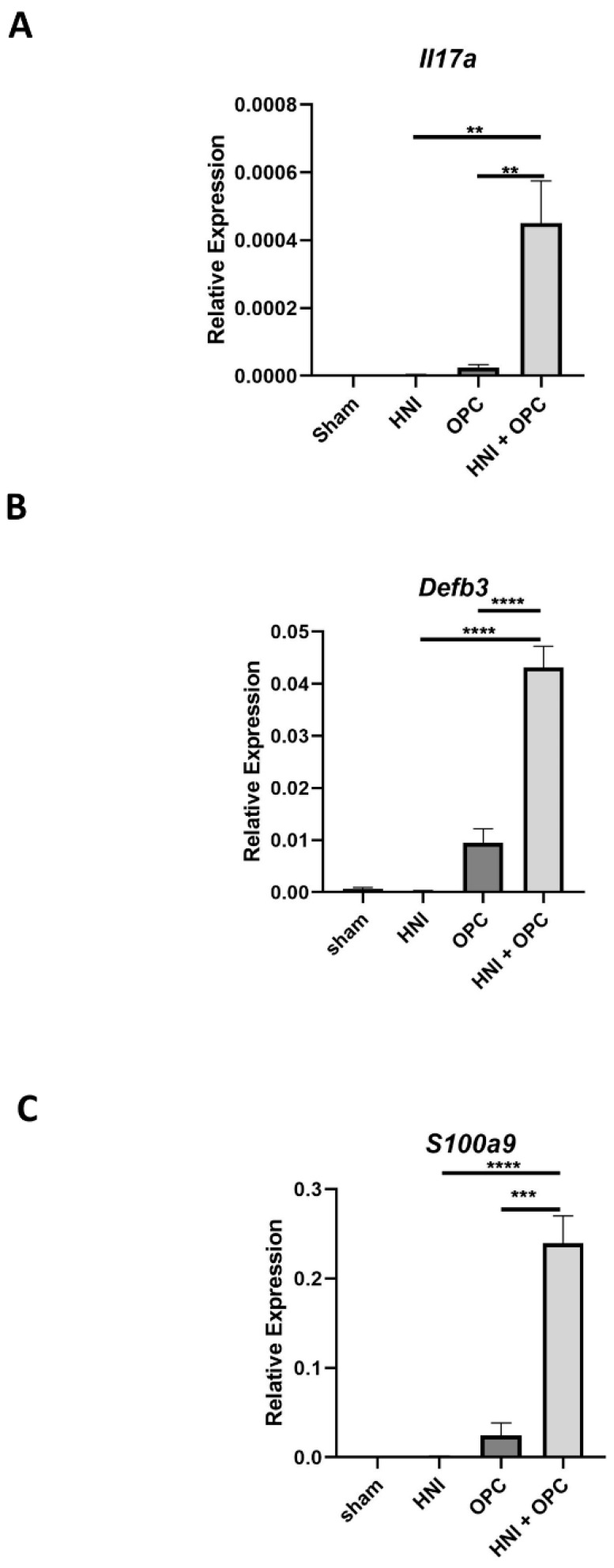
HNI enhances the IL-17-related response during OPC. (**A**–**C**) Expression differences relative to *GAPDH* of genes related to IL-17 antifungal responses. *Il17a, Defb3, S100a9* analyzed by one-way ANOVA with Tukey’s post hoc. (** *p* < 0.01, *** *p* < 0.001, **** *p* < 0.0001) n = 3–6 mice per group per experiment. Data represent 3 experimental repeats.

In order to understand how radiation damage leads to increased susceptibility to oral candidiasis, we exposed the head and neck regions of mice to a single dose of radiation that reliably induced oral mucositis using a clinical linear accelerator capable of IMRT delivery. We used this method previously to elucidate the protective attributes of IL-17RA during development of oral mucositis [28]. For this study, mice were irradiated in the head and neck targeted area on Day -1 with 22.5 Gy, and then were exposed on Day 0 to *C. albicans* sublingually (Figure 1A). The mice were weighed and observed daily, then tongue tissue was harvested on Day 4 post-infection. Both sham-irradiated and irradiated C57Bl/6J (WT) mice lost no weight by Day 4, which aligned with our previous findings when mice received head and neck irradiation (HNI) alone [28]. WT mice that were exposed to OPC without HNI lost weight initially and then regained by Day 4, while mice exposed to HNI and then OPC (HNI + OPC) lost weight by Day 2 and did not recover by Day 4 (Figure 1B). Toluidine blue staining of tissue is commonly used to visualize oral mucosal damage and the loss of barrier integrity caused by radiation [28]. Sham-HNI-, HNI-only-, and OPC-only-infected mice presented with very little to no damage on the tongue. In contrast, HNI + OPC mice had detectable ulcerative lesions on the tongue (~14% of the tissue staining with toluidine blue) (Figure 1C). This damage was associated with a decrease in mucosal thickness throughout the back and middle of the tongue in the HNI + OPC exposed mice (Figure S2 and Figure 1D). Aligning with the increased damage and loss of the mucosal layer, HNI + OPC mice had considerably higher fungal burden (1 × 10^5^ CFU/g tongue tissue) compared to all other groups, since immunocompetent WT mice clear C. albicans (0 CFU/g) by Day 4 post-infection (Figure 1E). Taken together, this indicated that mice are prone to more severe, prolonged OPC following irradiation by this method, which was associated with a loss of mucosal integrity. Since *Il17a* transcripts are induced by either radiation exposure or *C. albicans* infection, we next determined if HNI followed by OPC led to increased expression [14,28]. Indeed, the *Il17a* transcript levels were higher in the tongue tissue from the HNI + OPC group of mice compared to either the sham-infected mice or the WT mice that normally clear infection and downregulate *Il17a* expression by Day 4 (Figure 2A) [28]. Similarly, IL-17RA-regulated antifungal immune components *Defb3* and *S100a9* transcripts were highly induced in the oral cavities of mice exposed to radiation before OPC (Figure 2B,C). Transcripts of these three genes were not detected in WT mice 4 days post-HNI alone, which aligns with the progression of OM and expression of inflammatory cytokines at later time points post-irradiation exposure (Figure 2A–C) [28,30].

### 3.2. IL-17RA Signaling Protects against OPC following HNI

**Figure 3:**
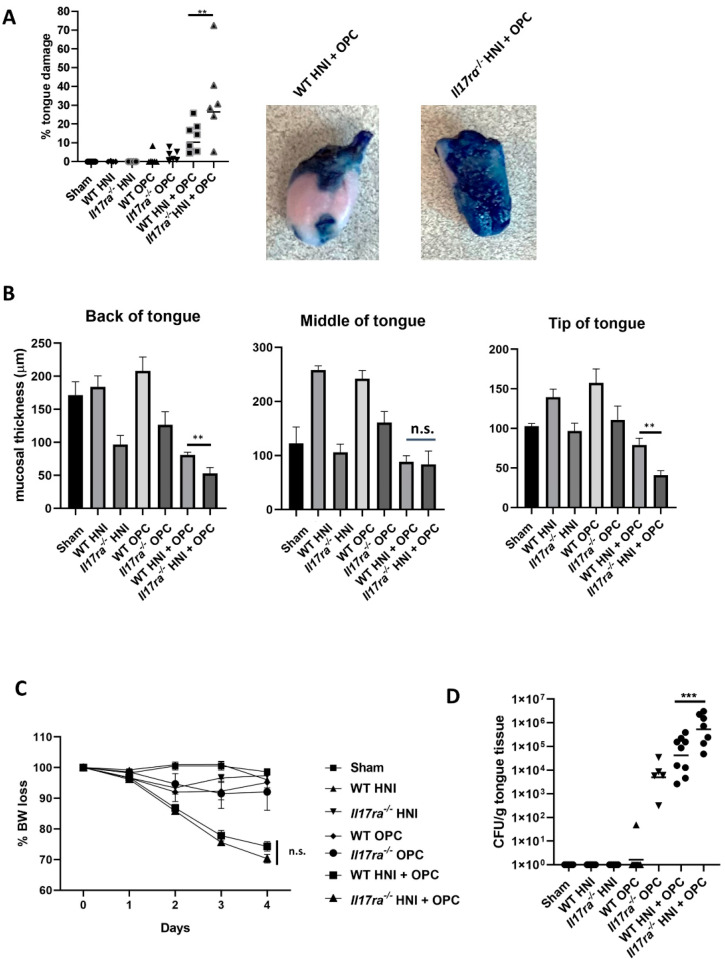
IL-17RA signaling protects against OPC following HNI. (**A**) Quantification of toluidine blue staining by determining the surface area of the tongue positive for blue staining compared to the total surface area of tongue for each mouse (n = 3 or more mice per group) on Day 4 post-infection. Analyzed by one-way ANOVA with Tukey’s post hoc. (**B**) Quantification of mucosal thickness on the tip, middle, and back of tongue. Measured from basal stem cell layer up to papillae using ImageJ software. Three mice were evaluated from each group, and three sections were analyzed per mouse. Investigators determining toluidine blue staining and measurement of mucosal thickness were blinded to treatment and mouse cohort. Analyzed by unpaired Student’s *t*-test. (**C**) All mice were weighed daily, and % body weight (BW) loss (gram) on each day of infection is indicated. (**D**) WT and *Il17ra*^−/−^ mice (n = 3–6 mice per group) were subject to HNI and 16 h later infected sublingually with *C. albicans* and on Day 4 CFU/g of tongue tissue was assessed in triplicate. Analyzed by Mann–Whitney U test. Data shown as geometric means. (** *p* < 0.01, *** *p* < 0.001) Data represent 3 experimental repeats.

Because *Il17a* transcript levels were elevated in the HNI + OPC condition, we next determined if IL-17/IL-17RA were still protective against fungal infection during this specific form of immunosuppression. Mice deficient in IL-17RA (*Il17ra*^−/−^) were subjected to HNI and infected with *C. albicans*. Larger ulcerative lesions were detected on *Il17ra*^−/−^ HNI + OPC tongues (~30%) compared to WT HNI + OPC mice (12%) (Figure 3A). The increased surface area of damage was also associated with a loss in mucosal thickness in areas of the tongue (Figure 3B). Both *Il17ra*^−/−^ and WT HNI + OPC mice lost weight compared to mice exposed to OPC alone (Figure 3C). The increased damage to the tongue tissue also correlated with fungal susceptibility, as *Il17ra*^−/−^ HNI + OPC mice had higher tissue fungal burden on Day 4 following infection compared to WT HNI + OPC mice and *Il17ra*^−/−^ +OPC-only mice (Figure S3 and Figure 3D). In all, these data reveal that IL-17 signaling is protective during OPC even when radiation exposure leads to increased levels of the proinflammatory cytokine.

### 3.3. Lack of IL-17RA Leads to Dissemination of C. albicans after HNI

**Figure 4:**
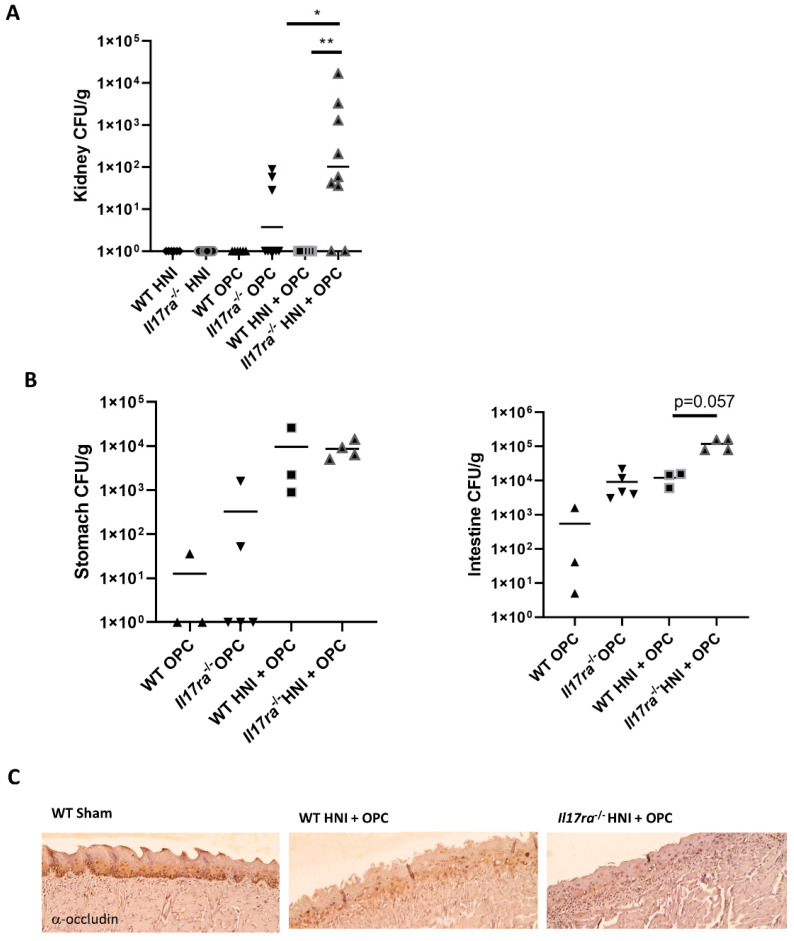
IL-17RA is required to prevent dissemination of *C. albicans* across the radiation-damaged oral mucosa. (**A**) WT and *ll17ra*^−/−^ (n = 3 or more mice per group) mice were subjected to HNI and 12–16 h later were infected sublingually with *C. albicans*. On Day 4 both kidneys were harvested, homogenized, and plated in triplicate. CFU/g of kidney tissue was determined. Analyzed by Mann–Whitney U test. Data shown as geometric mean. (**B**) On Day 4 both stomach and intestines were harvested, homogenized, and plated in duplicate. CFU/g of stomach and intestine tissue was determined (n= 3–6 mice per group). Analyzed by one-way Anova with Tukey’s post hoc. (**C**) Representative Occludin-staining of ulcer boundary region counterstained with hematoxylin. Images taken at 10x magnification. Images representative of 3 mice per group and 2 experimental repeats. (* *p* < 0.05, ** *p* < 0.01).

To test whether IL-17RA is involved in maintaining the oral epithelial barrier after radiation, we harvested kidneys on day 4 following infection to determine tissue fungal burden. *C. albicans* did not disseminate to the kidneys (0 CFU/g tissue) of WT mice even after a high dose of radiation (Figure 4A). In contrast, the kidneys from *Il17ra*^−/−^ HNI + OPC mice had a high level of *Candida* present compared to either WT HNI + OPC mice or *Il17ra*^−/−^ +OPC-only exposed mice (Figure 4A). Equivalent fungal burdens were detected in the stomach and intestines of *Il17ra*^−/−^ HNI + OPC mice compared to WT mice receiving the same treatment, likely due to the ingestion of *Candida* during the infection process (Figure 4B–D). To elucidate how IL-17RA may protect the oral mucosa against fungal dissemination, we assessed expression of the tight-junction protein occludin in the tongue tissue of mice exposed to HNI + OPC [20,21]. After irradiation exposure levels of occludin were decreased in WT mice compared to sham, while the expression of the protein was not detected in *Il17ra*^−/−^ HNI + OPC tongues (Figure 4C). This indicates a deficiency in the barrier of the mucosal layer following radiation when IL-17RA is absent, which may render the oral epithelia more permissive to *Candida* dissemination.

### 3.4. Heightened Neutrophil Recruitment in IL-17RA-Deficient Mice in Response to OPC after HNI

**Figure 5:**
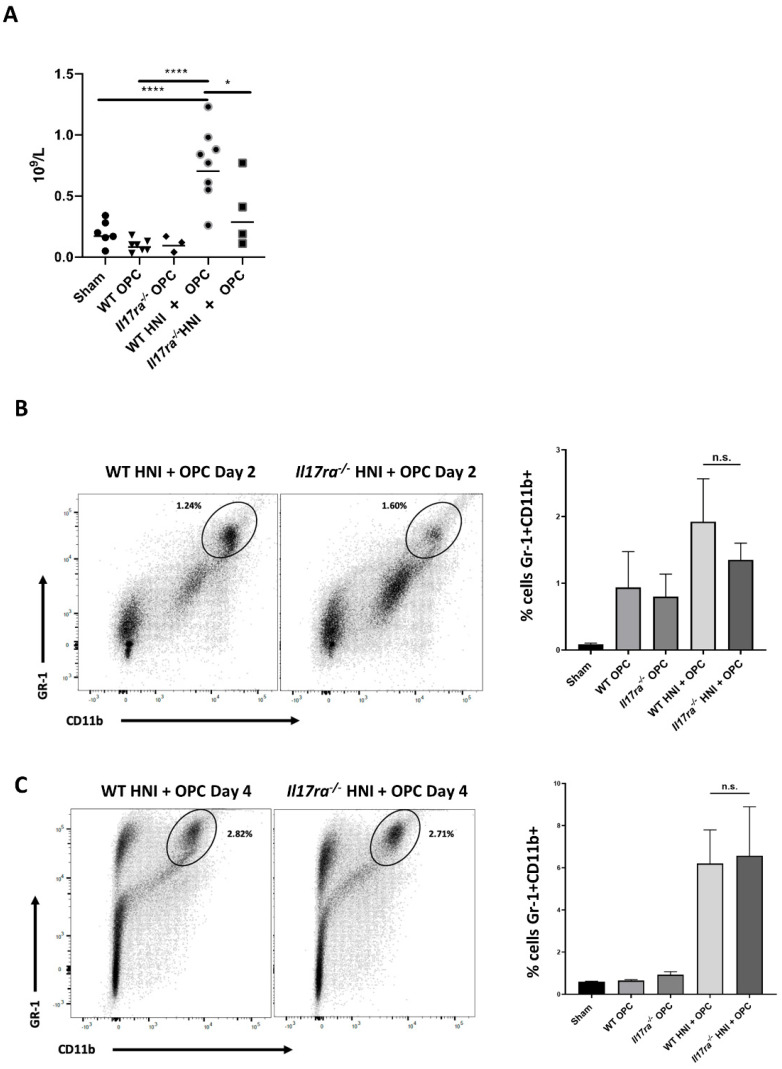
Heightened neutrophil recruitment in IL-17RA-deficient mice in response to OPC after HNI. (**A**) Cardiac puncture was performed to harvest blood from each mouse and the number of circulating neutrophils measured on Day 4 post-infection (n = 3 or more mice per group). (**B**,**C**) Flow cytometry performed on whole tongues harvested Day 2 and 4 after subjecting mice to OPC and HNI + OPC, followed by quantification of Gr-1 + CD11b+ neutrophils. (n = 3–6 mice per group). Analyzed by one-way ANOVA with Tukey’s post hoc. (* *p* < 0.05, **** *p* < 0.0001). Data represent 3 experimental repeats.

**Figure 6:**
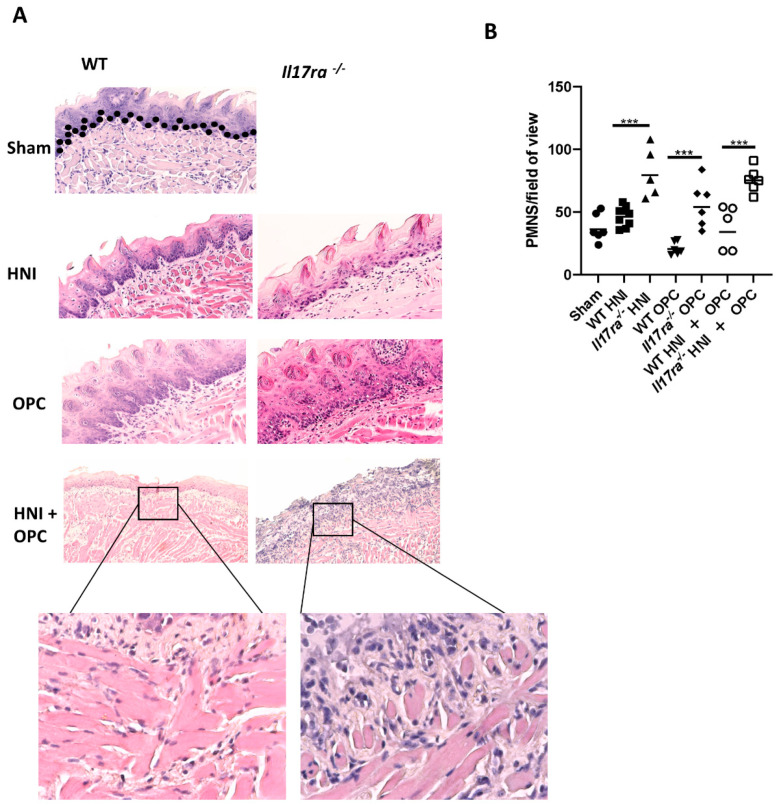
Increased neutrophil influx into tissue damaged by irradiation in the absence of IL-17RA. (**A**) Staining by hematoxylin and eosin of sectioned tongues and quantification of neutrophils on day 4 (n = 3–6 mice per group). Top Images taken at 20× magnification, inset images taken at 40× magnification. (**B**) Neutrophil quantification analyzed by one-way ANOVA with Tukey’s post hoc. Evaluators blinded to the treatment group and mouse cohort analyzed the tissue sections. (*** *p* < 0.001) Data represent 3 experimental repeats.

Neutrophils are important for protection against *C. albicans* [2,35]. Mice lacking IL-17RA have insufficient recruitment of neutrophils into the tongue tissue during infection, partially contributing to the increased susceptibility of *Il17ra*^−/−^ mice to OPC compared to WT mice [9,14]. Even so, when *Il17ra*^−/−^ mice are exposed to HNI, more neutrophils are present in the damaged oral mucosa due to the dysregulation of other cytokines involved in neutrophil recruitment in the absence of IL-17RA [28]. Since *Il17ra*^−/−^ mice have deficient levels of neutrophils when exposed to OPC alone yet have excess neutrophils when exposed to HNI, we next determined how HNI + OPC skews the neutrophil response. Both WT and *Il17ra*^−/−^ mice exposed to either HNI or OPC only had circulating neutrophil levels comparable to sham mice (Figure 5A). WT mice exposed to HNI + OPC had increased neutrophils in circulation, while *Il17ra*^−/−^ mice did not have a similar increase in neutrophils in the blood after radiation and infection (Figure 5A). Next, we determined the neutrophil population in the whole tongue tissue from each mouse. On days 2 and 4 post-infection both *Il17ra*^−/−^ HNI + OPC mice and WT HNI + OPC mice had similarly elevated Gr-1 + CD11b+ neutrophils in tissue compared to mice that received either treatment alone (Figure 5B,C). We then histologically evaluated the polymorphonuclear cells (PMNs) that migrated into the damaged tongue tissue on Day 4 after infection. This allowed visualizing the neutrophils within the sub-basal and supra-basal layers of the oral mucosa after HNI + OPC. Unlike with the whole tongue tissue, *Il17ra*^−/−^ mice exposed to HNI + OPC had considerably more neutrophils in the sub- and supra-basal region of the damaged oral mucosa compared to WT HNI + OPC mice, which had lower neutrophil counts (Figure 6A,B). The levels of neutrophils in HNI-only mice were similar to sham-HNI mice. This time point was 5 days post-HNI, while the peak of damage, and thus neutrophil influx, is on Day 11 after irradiation if there is no exposure to OPC (Figure 6A,B).

### 3.5. Inadequate Anti-Candida Neutrophil Response in IL-17RA-Deficient Mice

**Figure 7:**
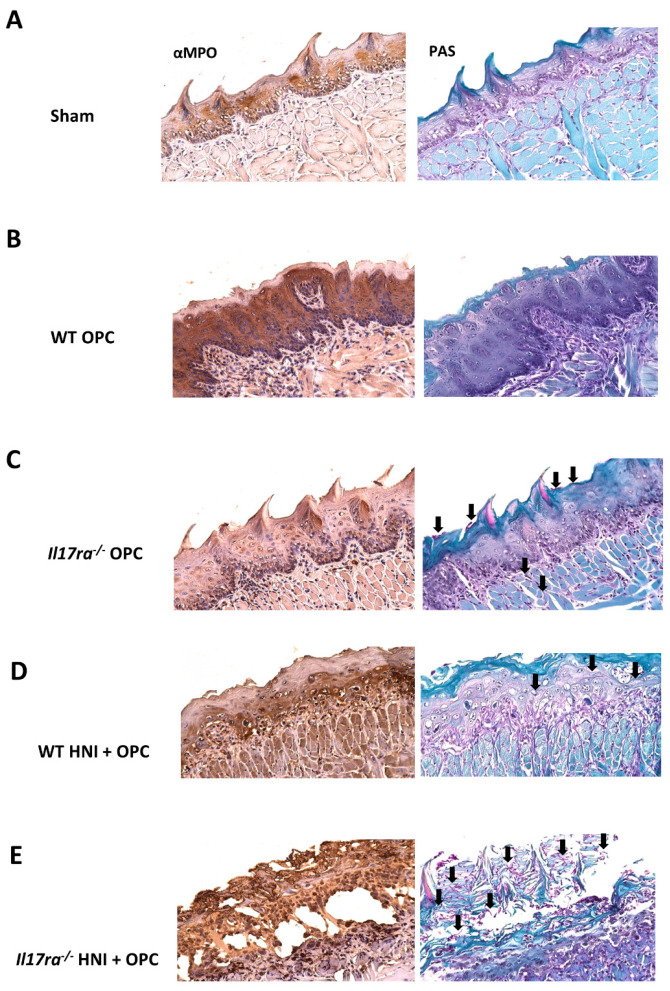
Higher MPO levels throughout the epithelial layers of the tongue tissue in the presence of *C. albicans*. (**A**–**E**) Representative MPO-staining of ulcer boundary region counterstained with hematoxylin. Serial section was stained with PAS to visualize *C. albicans* and counterstained with Fast Blue. Images taken at 20× magnification. Arrows indicate examples of *C. albicans*. Images representative of 3 mice per group and 3 experimental repeats.

**Figure 8:**
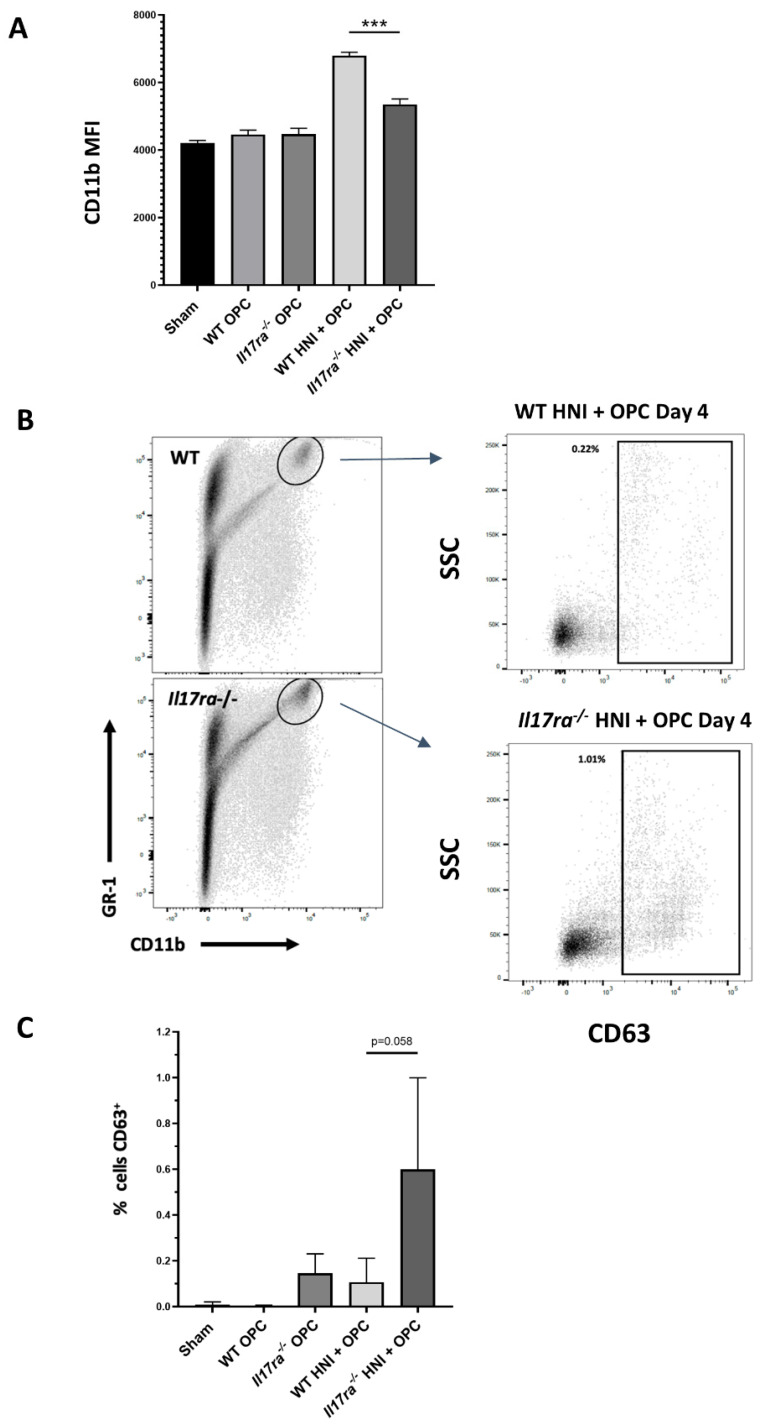
HNI exposure leads to oral neutrophil defects. (**A**) Quantification of flow cytometry results showing phagocytosis of GFP-*Candida albicans* by Gr-1+ neutrophils after subjecting mice to OPC and HNI + OPC (n = 3 mice per group) (**B**,**C**) Expression differences relative to *GAPDH*. Analyzed by ANOVA with Tukey’s post hoc. (n = 3 mice per group). (*** *p* < 0.001). Data representative of at least 2–3 experimental repeats.

**Figure 9:**
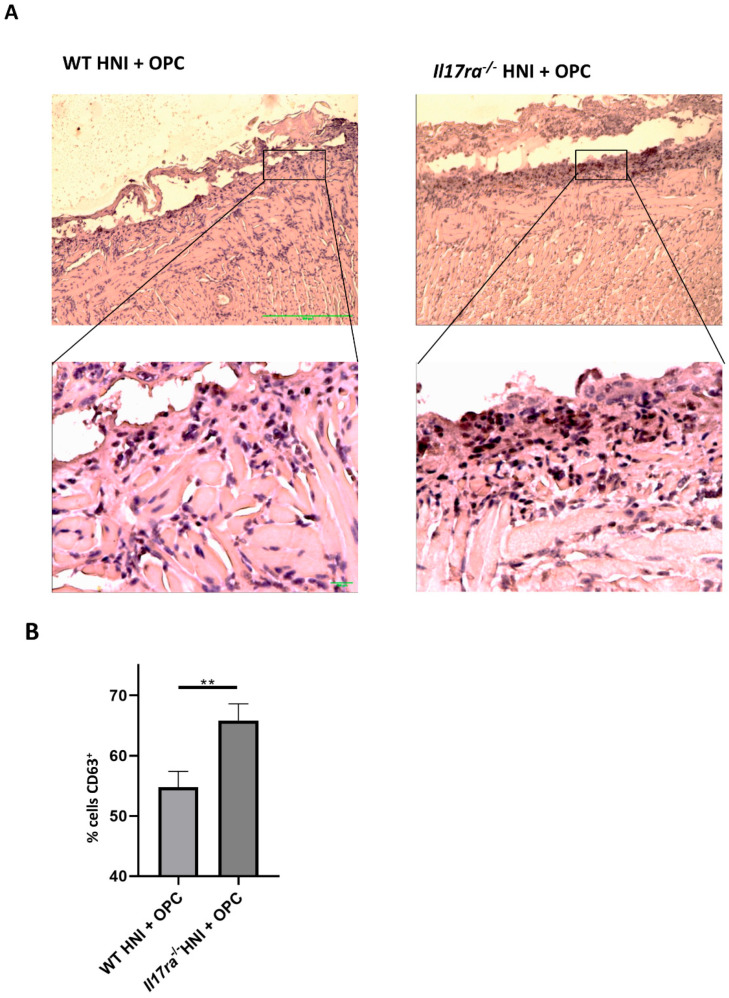
Enhanced CD63 expression in irradiated tongue tissue in the absence of IL-17RA. (**A**) Representative immunohistochemical staining for CD63 in the ulcer boundary region counterstained with hematoxylin. Images representative of 2 mice per group. Top Images taken at 10× magnification, bottom images taken at 40× magnification. (**B**) CD63+ neutrophil quantification analyzed by Student’s *t*-test. Each data point represents the number of CD63+-stained neutrophils in 10 fields of view for each mouse (n = 2 mice per group). Evaluators blinded to the treatment group and mouse cohort analyzed the tissue sections. (** *p* < 0.01).

**Figure 10:**
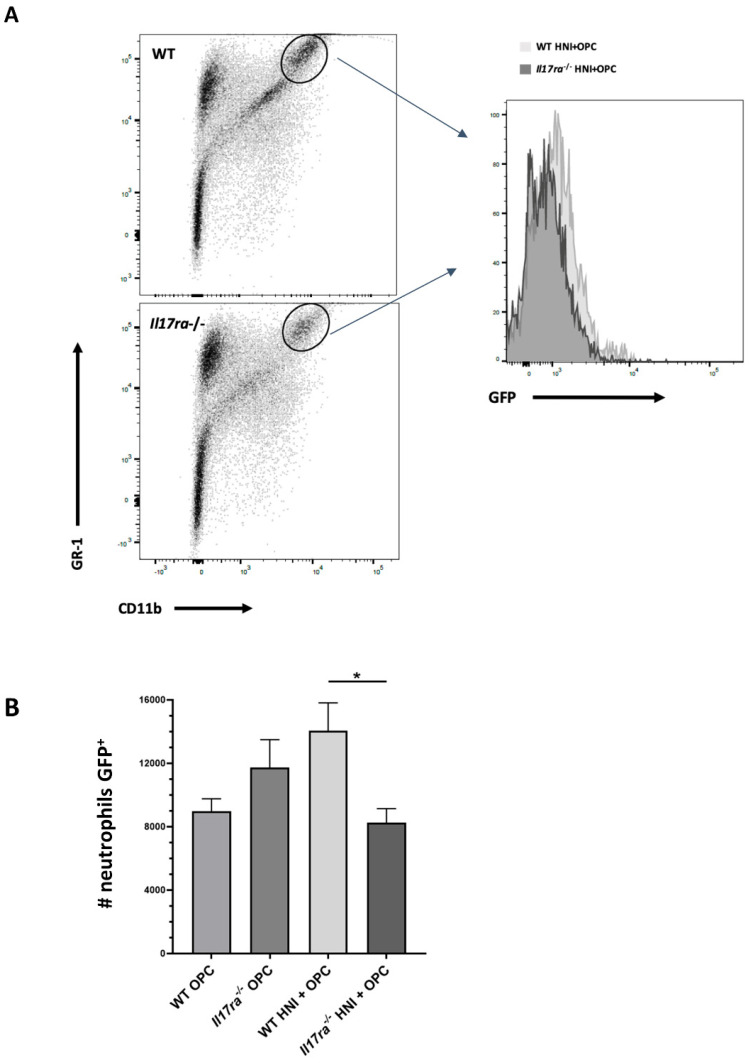
Decreased phagocytic activity by neutrophils in IL-17RA-deficient mice in response to OPC after HNI. (**A**,**B**) Flow cytometry was performed on whole tongues harvested Day 4 after exposing mice to OPC and HNI +OPC with a GFP-expressing *Candida albicans* strain. Data represent the number of GR1 + CD11b+ neutrophils positive for GFP. Analyzed by one-way ANOVA with Tukey’s post hoc. (n = at least 3 mice per group). (* *p* < 0.05). Data represent 2 experimental repeats.

Even though *Il17ra*^−/−^ mice had excess neutrophils in the oral mucosa after HNI + OPC, the mice still had high fungal burden, indicating the neutrophils may not be functionally competent after radiation exposure. As a readout of neutrophil activity, we assessed myeloperoxidase (MPO) levels in the tongue tissue using immunohistochemistry. WT mice exposed to only OPC had higher MPO production compared to infected *Il17ra*^−/−^ mice, which aligns with the high fungal burden in *Il17ra*^−/−^ compared to immunocompetent WT mice that clear infection by this time point (Figure 7A–C). WT mice subjected to HNI + OPC showed lower levels of MPO compared to immunocompetent WT mice that cleared OPC (Figure 7B,D). When exposed to HNI + OPC, *Il17ra*^−/−^ mice had higher MPO levels throughout the epithelial layers of the tongue tissue that coincided with the presence of *C. albicans* in the same areas visualized by PAS staining compared to WT HNI + OPC or *Il17ra*^−/−^ OPC mice (Figure 7C–E). Since higher MPO levels in the tissue may represent the presence of more neutrophils, not necessarily a functional difference in the cells there, we used flow cytometry to investigate phenotypic differences in neutrophils after radiation exposure. Upon activation, neutrophils upregulate the surface expression of the adhesion molecule CD11b for entry into inflamed tissue [36]. While there were more neutrophils in the whole tongue of both WT and *Il17ra*^−/−^ mice after HNI + OPC on Day 4 (Figure 5C), the Gr-1+ cells were expressing less CD11b on a per cell basis in *Il17ra*^−/−^ mice compared to WT mice, indicating an issue with activation in the absence of IL-17RA (Figure 8A). There was also a trend for the neutrophils from *Il17ra*^−/−^ HNI + OPC mice to express higher levels of the degranulation marker CD63 compared to WT HNI + OPC mice (Figure 8B,C). Next, we focused on the areas of damage where *Candida* was present. Immunohistochemistry showed that the *Il17ra*^−/−^ HNI + OPC mice had more PMNs staining positive for CD63 than WT HNI + OPC (Figure 9A,B). Even though there were more neutrophils in the tongue tissue of *Il17ra*^−/−^ mice exposed to HNI + OPC (Figure 6B), these cells had decreased capacity to engulf *C. albicans* after radiation compared to WT mice (Figure 10A,B), which correlated with the high fungal burden in mice lacking IL-17RA (Figure 3D). Additionally, with irradiation, *S100a9* was reduced in the absence of IL-17RA, which correlates with neutrophils that have enhanced oxidative metabolism and reduced apoptotic ability, perhaps contributing to the amplified tissue damage in the HNI + OPC *Il17ra*^−/−^ mice (Figure S4A) [37,38]. S100a8/9 (calprotectin) can also kill *C. albicans* directly, and decreased production of the antimicrobial peptide may at least partially account for the overgrowth of the fungus when IL-17RA is absent. Furthermore, *Il17ra*^−/−^ mice had enhanced levels of *Mmp9*, which encodes for matrix metalloproteinase 9 (MMP9) (Figure S4B). 

The overexpression of MMP9 promotes migration of neutrophils into tissue and can lead to uncontrolled damage through production of free radicals and rapid release of nitric oxide, potentially contributing to the lack of proper healing in the HNI + OPC *Il17ra*^−/−^ mice [39]. Both *Il1a* and *Illb* transcripts were upregulated in the *Il17ra*^−/−^ HNI + OPC mice compared to WT (Figure S4C,D) and may account for the increased neutrophil influx in the tongue tissue, which is similar to the role of IL-1α and IL-1β at the peak of damage during oral mucositis caused by HNI-only in *Il17ra*^−/−^ mice [28]. Overall, in the absence of IL-17RA there was an accumulation of functionally aberrant neutrophils in the irradiated tissue that were contributing to the dysregulated inflammatory response and overgrowth of *C. albicans*.

## 4. Discussion

A better understanding of IL-17 at the intersection of radiation damage and oral infection is warranted. Post-HNI damage to the oral mucosal layer and saliva production can perturb the oral flora and lead to an increased incidence of infections, including OPC and herpes simplex virus (HSV) infection [3,5,40]. The existing therapies for OM are inadequate and thus far only mitigate symptoms, not prevent severe damage [41,42,43,44]. Since IL-17 is involved in both the progression of the inflammatory response post-irradiation and antifungal immunity to *Candida,* the next step was to determine how radiation perturbs IL-17-mediated responses during OPC.

As expected, radiation exposure led to increased susceptibility of immunocompetent mice to OPC (Figure 1) [45,46,47]. The condition of HNI + OPC led to elevated levels of *Il17a* transcript expression in the oral mucosa compared to just HNI or OPC alone. Even though IL-17 is beneficial during OM and OPC individually, it was not straightforward that IL-17 would still be protective when there is both radiation damage and infection present [11,14,16,28]. Yet, *Il17ra*^−/−^ mice had higher fungal burden after radiation exposure compared to WT mice. What was unique was the large influx of neutrophils in the absence of IL-17RA. Although radiation resulted in increased neutrophils in *Il17ra*^−/−^ *Candida* infected tongues, these cells were not protective and had defects in activation and phagocytic capacity.

The protective nature of IL-17 in antifungal immunity is generally accepted [10]. However, questions remain on the role of IL-17 in control of the neutrophil response, and the relative importance of other proinflammatory cytokines in the regulation of neutrophils in different forms of candidiasis [9,35,48]. We now shed light on the role of IL-17 in mediating neutrophils during the excessive inflammation caused by radiation-induced OPC. In the absence of IL-17RA, there were enhanced levels of neutrophils in the HNI + OPC condition, yet these cells were unable to control fungal growth. This suggests that radiation is not only causing physical destruction of the epithelial barrier but also that immune mediators are skewed by radiation to increase susceptibility to candidiasis. We previously found that not all proinflammatory cytokines are broadly upregulated when WT mice are exposed to HNI alone. Instead, post-irradiation, IL-17 is induced and required for healing and reconstitution of the oral mucosa [28]. When IL-17RA signaling is abrogated, radiation damage leads to the accumulation of neutrophils. Yet, this additional recruitment does not result in clearance of *C. albicans* but rather excess inflammation and damage. The neutrophils from *Il17ra*^−/−^ mice show skewed expression of activation markers such as CD11B and increased production of MPO, CD63, *MMP9*, and *S100a9*, which could contribute to pathogenic inflammation and delayed healing in the absence of IL-17RA [49]. Future studies will discern why these neutrophils that are recruited to the oral cavity post-HNI (and therefore not exposed to radiation directly) are ineffective against *Candida*.

Presumably, radiation damage leads to functional differences in other immune mediators that normally require IL-17RA for homeostasis. Since, after radiation, the oral immune niche is disrupted, the contribution of other cytokines implicated in antifungal responses, such as IL-23, IL-22, and IL-1, will need to be established as well [14,48,50,51]. We did not find a major role for IL-22 during the development of HNI-induced OM (data not shown and [28]). Yet, we cannot exclude the possibility that IL-22 is involved in the response to *C. albicans* post-irradiation. Additionally, IL-1/IL-1R have been implicated in driving the neutrophil response in forms of candidiasis, as well as other fungal infections [48,52,53,54,55,56]. Here, we show that increased neutrophils are pathophysiological during OPC, which aligns with the understanding of these cells during VVC and supports the dynamic nature of tissue-specific neutrophils. Future studies will need to address the interplay between IL-17 and other cytokines in control of the inflammatory response post-irradiation and the individual contributions of these mediators to antifungal immunity. We also did not explore the cellular source of IL-17 during HNI + OPC. Normally, during acute OPC, innate lymphocytes, not neutrophils, make IL-17 [13,57]. Yet, during fungal keratitis, neutrophils produce IL-17, which acts in an autocrine manner in concert with other cytokines to further activate neutrophils in the eye [51,58]. Further studies will determine if neutrophils produce IL-17 in the radiation disrupted oral mucosa.

In general, *C. albicans* is contained within the oral cavity, and the fungus does not breach the mucosae to establish a disseminated infection [10]. Yet, radiation-induced mucosal damage can allow oral microbes, including *Candida*, into circulation through the lesions caused by this therapy [6,7,44]. Here, in the absence of IL-17RA, *Candida* was detected at higher levels in the kidneys, even though WT mice that received HNI + OPC presented with overt damage to the tongue tissue as well (Figure 1C). This aligned with decreased occludin expression in the oral mucosa of *Il17ra*-/- mice, indicating a barrier defect in the absence of IL-17RA. This is similar to the role of IL-17 in the maintenance of the gut epithelia [20,21]. During experimental OPC, large amounts of *C. albicans* yeast are placed in the oral cavity, so mice will ingest some of the cells, and fungal burden can be detected in the stomach and intestines. Therefore, we cannot exclude that *C. albicans* may be gaining access to peripheral organs across the lower intestinal tract, rather than through the oral mucosa specifically. Yet, radiation exposure does lead to increased dissemination to the kidneys in the absence of IL-17RA. We can speculate this movement is through the damaged oral cavity though, since the radiation is targeted to the head/ neck regions and the lower gastrointestinal tract is not exposed. It will be interesting to compare targeted HNI to total-body irradiation and ascertain how the immune environment of the entire orogastrointestinal tract is affected when all hematopoietic and non-hematopoietic compartments are irradiated.

In this study, we used a linear accelerator with the limitation that infected mice are not allowed in the patient treatment areas. Therefore, it was only possible to irradiate first then induce OPC in the mice. Even though necessary, this experimental set-up may not entirely translate since *Candida* is often part of the normal human oral flora and would be present during radiation [1]. *C. albicans* is not a normal commensal in specific pathogen free (SPF) mice, but we exposed mice to the fungus only 12 to 15 h after HNI [33]. This ensures that the addition of *Candida* is still during the initiation phase of OM associated with initial DNA damage, not when OM further progresses then peaks eleven to twelve days later [28,40]. Since overt OM lesions heal by day 13 post-HNI in our mice, it is prudent to shift the schedule and determine the role of IL-17 in fungal infection that is present during peak radiation damage and the healing phase of the mucosae [28].

The absence of IL-17RA signaling during radiotherapy may not be exclusive to genetically modified mice and the relatively small number of identified humans with polymorphisms related to the pathway [12]. These findings could also be relevant to individuals receiving therapies targeting the Th17/IL-17 axis for autoimmune conditions, including psoriasis. Patients receiving these IL-17-related inhibitors have an increased risk of developing *Candida* infections, especially oropharyngeal and esophageal candidiasis [24]. In the HNI-induced OM model, when WT mice were administered α-IL-17A antibodies, more severe damage to the oral cavity developed [28]. The next step will be to determine if IL-17/IL-17RA blockade leads to increased fungal susceptibility after HNI. These findings will be key to understanding whether patients receiving anti-IL-17-related therapies will need to adjust their treatment regimens if faced with a cancer diagnosis. In these cases, treatments for the malignancies, especially head and neck squamous cell carcinomas (HNSCC), will need to be coordinated with therapeutics for the existing autoimmune conditions. Overall, we shed light on the role of IL-17 in antifungal immune responses when radiation causes severe damage and inflammation in the oral mucosa. The cytokine is necessary to temper neutrophil accumulation, and in the absence of IL-17RA, the neutrophils that are recruited to the oral cavity are not functional against *Candida*. These findings will allow for better treatment plans that consider the malignancy and the complications that arise from the radiotherapy.

## Data Availability

Not applicable.

## Supplementary Materials

The following supporting information can be downloaded at: https://www.mdpi.com/article/10.3390/jof8050495/s1.
