# Supplementary material for: Radiation Exposure Perturbs IL-17RA-Mediated Immunity Leading to Changes in Neutrophil Responses That Increase Susceptibility to Oropharyngeal Candidiasis
Source: J Fungi (Basel). 2022 May 10;8(5):495. doi: 10.3390/jof8050495 (PMC9144824; doi:10.3390/jof8050495)
Supplement: Supplementary file 1 [file jof-08-00495-s001.zip › jof-1632674-supplementary.pdf]

Saul-McBeth et al. 2022 Supplemental Figure S1

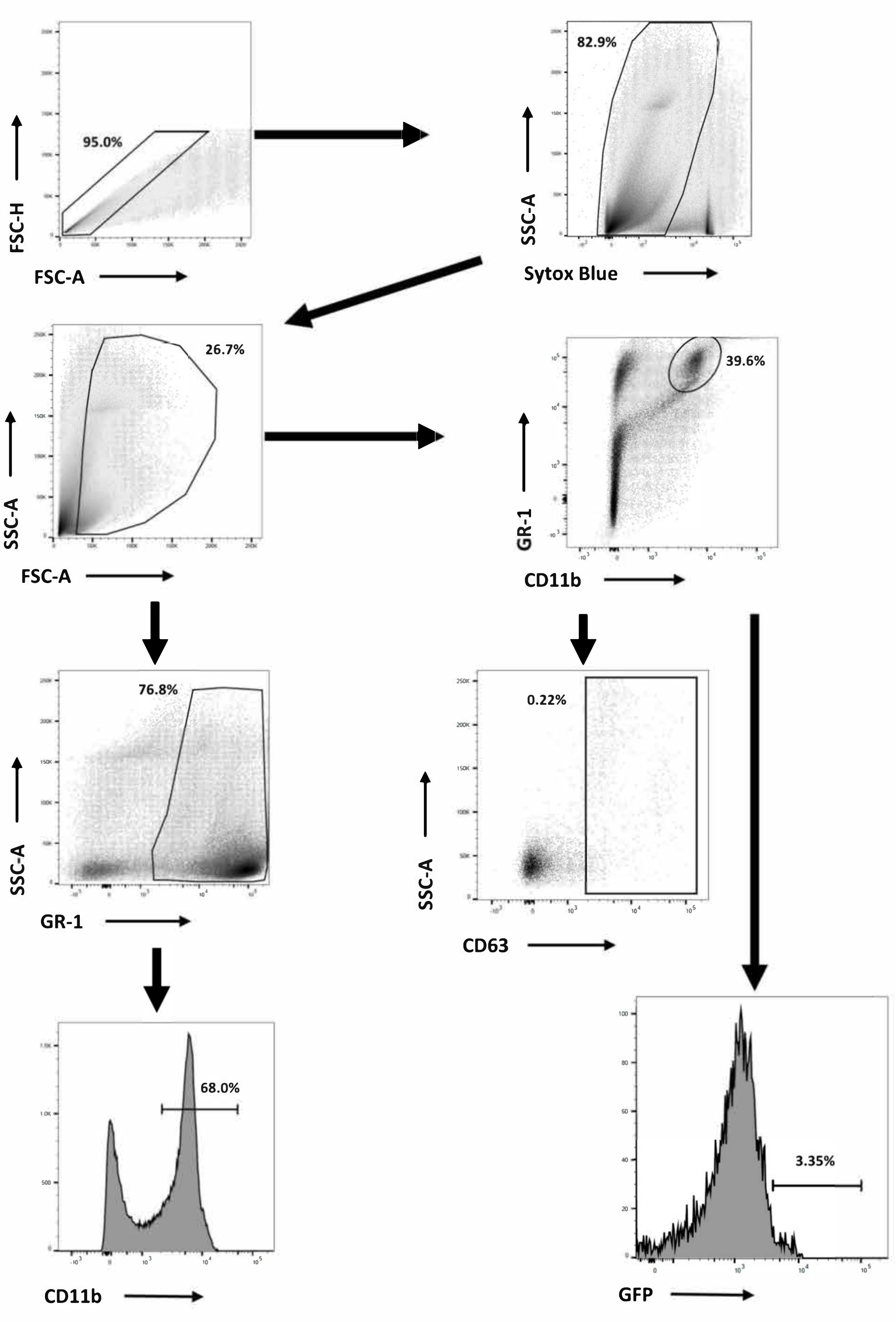

Supplemental Figure S1 Flow cytometry gating strategy Initially, doublets are removed by FSC-A vs. FSC-H gating followed by live cell gating using Sytox Blue vs. SSC-A. Next, a general leukocyte gate is applied using SSC-A vs. FSC-A. For analysis of CD63 and GFP signals, a double positive gate of GR-1+CD11b+ is used, followed by respective CD63 and GFP gating. For CD11b MFI analysis, a gate of GR-1+ cells (vs. SSC-A) is used, followed by a CD11b+ gate and Geometric MFI statistics.

Saul-McBeth et al. 2022 Supplemental Figure S2

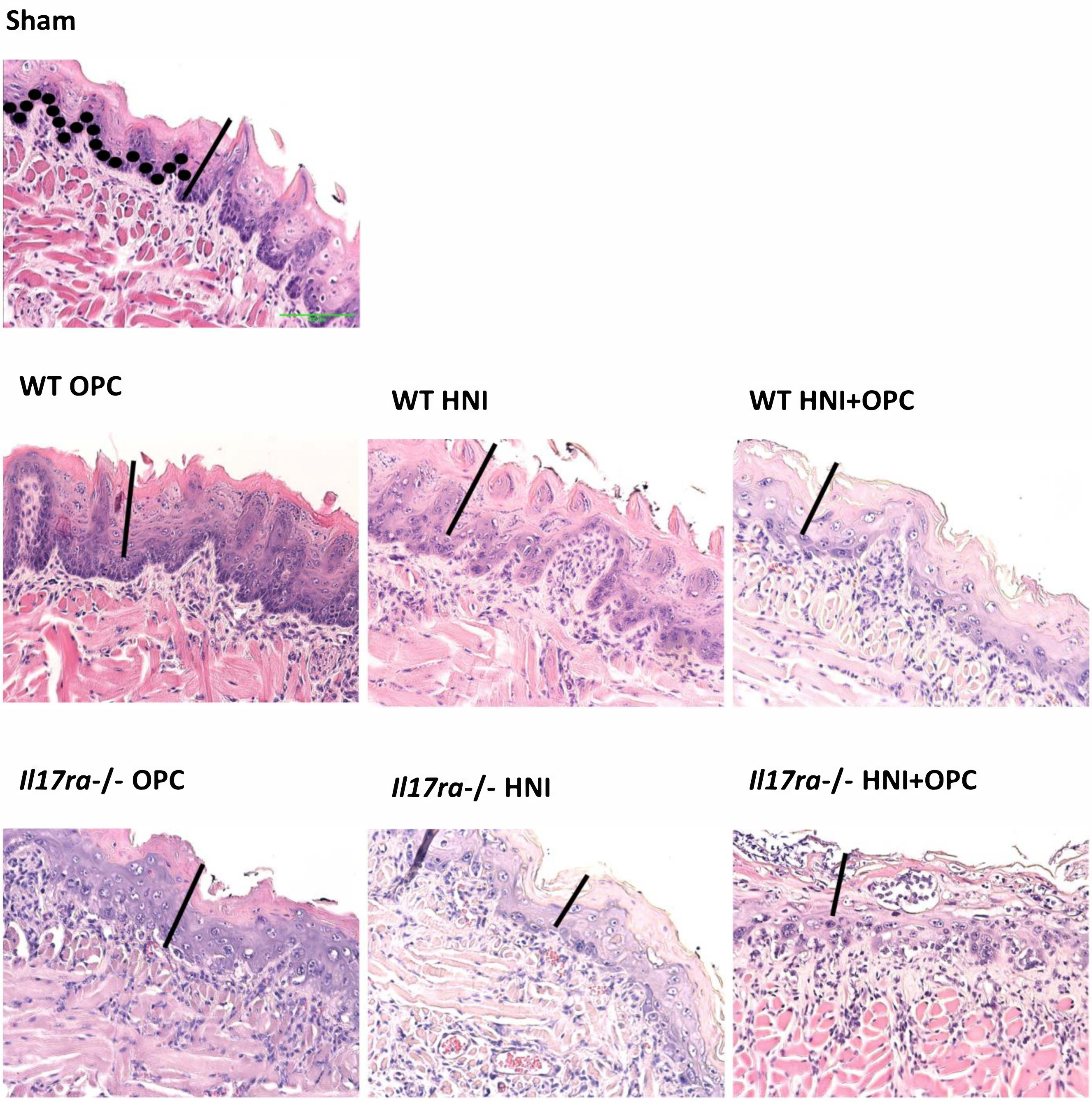

Supplemental Figure S2 Mucosal Thickness of Supra-basal region of tongue tissue Representative images of mucosal thickness determination in ImageJ. Dotted line represents the basal stem cell layer. Solid line is distance measured from basal stem cell layer up to superficial region of the tongue tissue through to the tip of the papillae as present in the Sham tissue.

Saul-McBeth et al. 2022 Supplemental Figure S3

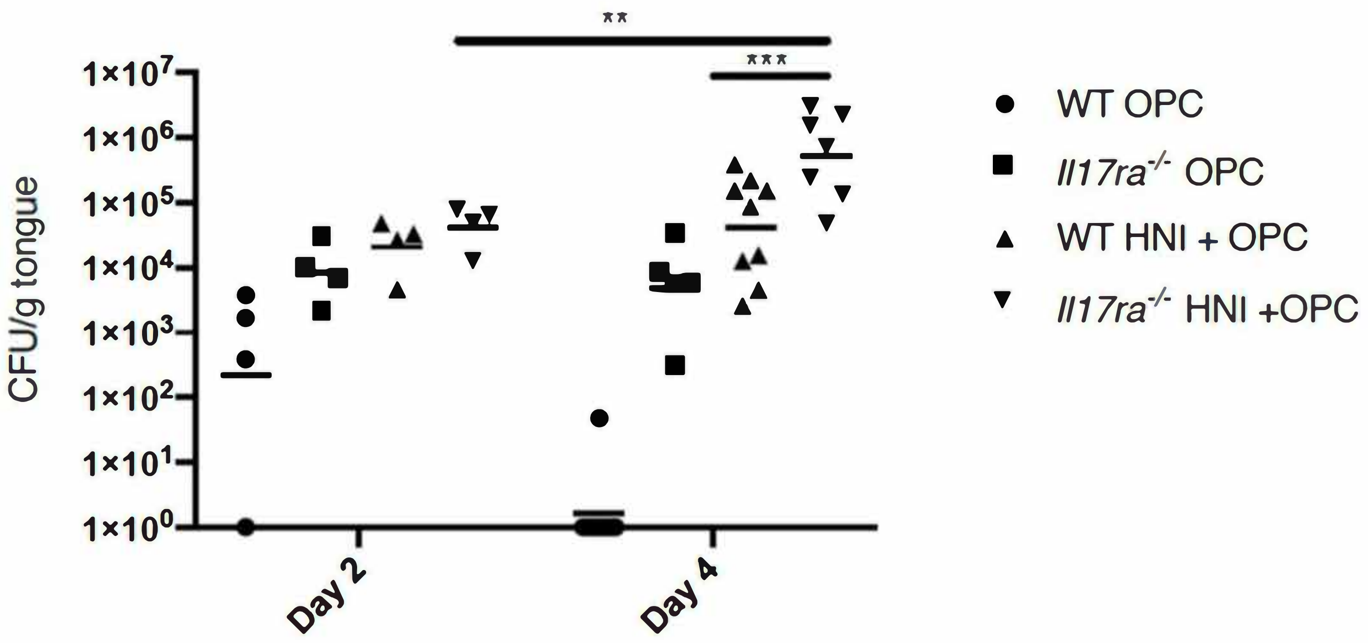

Supplemental Figure S3 Fungal susceptibility on Day 2 and Day 4 post-infection WT and *Il17ra*$^{-/-}$ mice (n= 3 or more mice per group) were subject to HNI and 16 hours later infected sublingually with *C. albicans* and on Day2 and 4 CFU/g of tongue tissue was assessed in triplicate. Analyzed by Mann-Whitney U test. Data shown as geometric means. (**P < 0.01, ***P < 0.001) Data represents 3 experimental repeats.

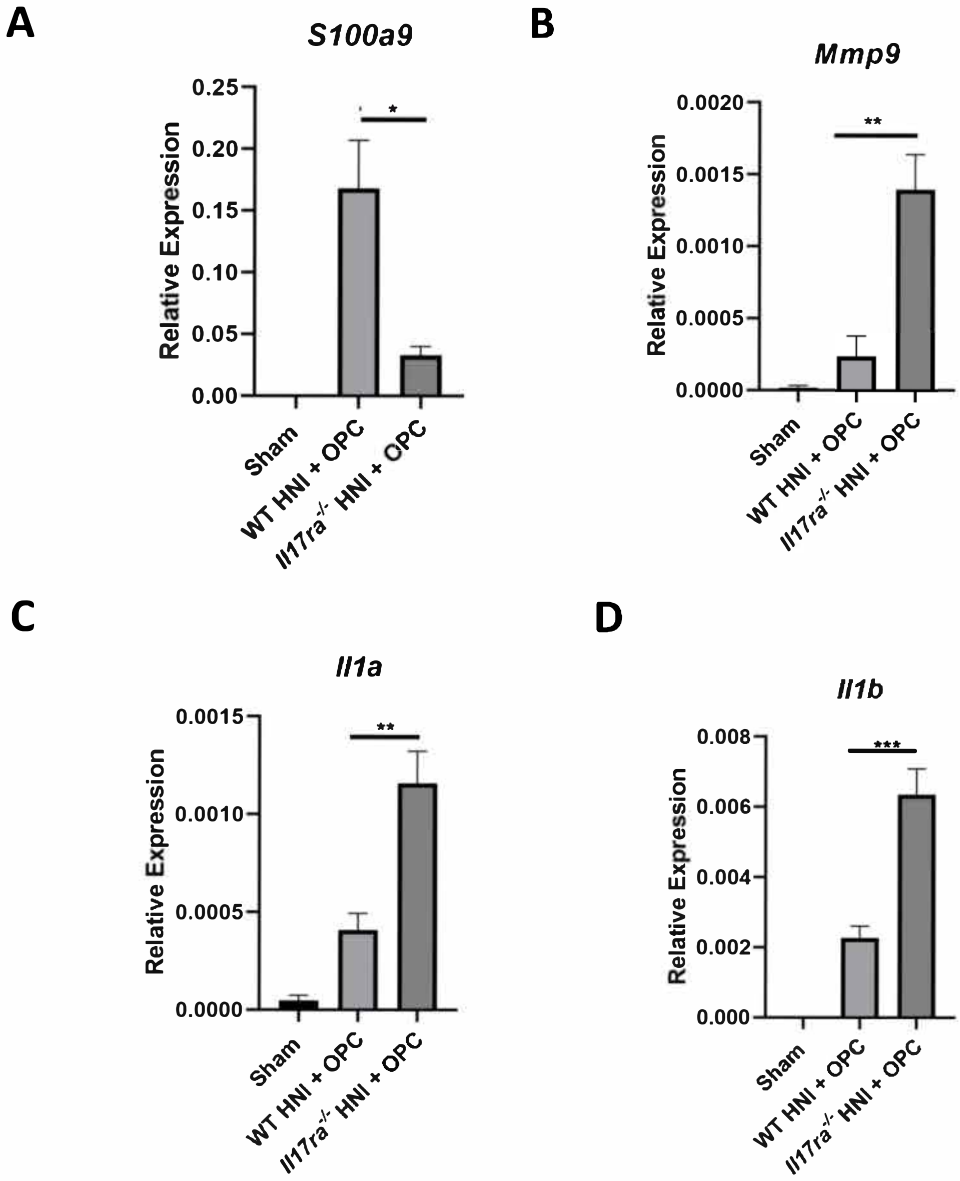
A
S100a9
Relative Expression
0.25
0.20
0.15
0.10
0.05
0.00
*
Sham
WT HNI + OPC
Il17ra-/- HNI + OPC
B
Mmp9
Relative Expression
0.0020
0.0015
0.0010
0.0005
0.0000
**
Sham
WT HNI + OPC
Il17ra-/- HNI + OPC
C
Il1a
Relative Expression
0.0015
0.0010
0.0005
0.0000
**
Sham
WT HNI + OPC
Il17ra-/- HNI + OPC
D
Il1b
Relative Expression
0.008
0.006
0.004
0.002
0.000
***
Sham
WT HNI + OPC
Il17ra-/- HNI + OPC

Supplemental Figure S4 Gene expression changes on Day 4 post-infection Expression differences relative to *GAPDH*. Analyzed by ANOVA with Tukey's post hoc. (n=3 mice per group). (*P < 0.05, **P < 0.01, ***P < 0.001). Data representative of at least 2-3 experimental repeats.
